# Cost-Effective Screening for Trichomoniasis

**DOI:** 10.3201/eid0807.010543

**Published:** 2002-07

**Authors:** Jane R. Schwebke

**Affiliations:** University of Alabama at Birmingham, Birmingham, Alabama, USA

**To the Editor:** I read with interest a recent article in your journal, “*Trichomonas* *vaginalis*, HIV, and African Americans” (1), and I commend the authors’ suggestion to implement screening and reporting of trichomoniasis for high-risk populations.

In the article, a cost-effective screening approach is mentioned, which includes culturing only for those women whose wet-mount tests are negative. In 1999, my colleagues and I reported on the validity of this method for diagnosing trichomoniasis in women (2). During our study, an additional vaginal swab was collected during the pelvic examination and placed into a glass tube. If the wet mount was negative, this swab was later added to a culture pouch for *T.* *vaginalis*. We found no statistically significant difference in the sensitivity of this method compared with that of adding swabs immediately to pouches at bedside. This method of delaying the second test until the results of the first test are known should be considered in screening women for trichomoniasis, especially in high-prevalence populations.
